# Prevalence of metabolic syndrome and its association with rapid weight loss among former elite combat sports athletes in Serbia

**DOI:** 10.1186/s12889-024-17763-z

**Published:** 2024-01-22

**Authors:** Nemanja Maksimovic, Ognjen Cvjeticanin, Carlo Rossi, Marko Manojlovic, Roberto Roklicer, Antonino Bianco, Attilio Carraro, Damir Sekulic, Aleksandra Milovancev, Tatjana Trivic, Patrik Drid

**Affiliations:** 1 Sport and Exercise Research Unit, Department of Psychology, Educational Science and Human Movement, University of Palermo, Via Giovanni Pascoli 6, Palermo, 90144 Italy; 2 Faculty of Sport and Physical Education, University of Novi Sad, Novi Sad, 21000 Serbia; 3 Centro Medico Di Fisioterapia “Villa Sarina”, Alcamo, 91011 Italy; 4 Faculty of Education, Free University of Bozen-Bolzano, 39042 Brixen–Bressanone, BZ Italy; 5 Faculty of Kinesiology, University of Split, Split, 21000 Croatia; 6 Faculty of Medicine, University of Novi Sad, Novi Sad, Serbia; 7 Institute of Cardiovascular Diseases of Vojvodina, Sremska Kamenica, Serbia

**Keywords:** Metabolic pathologies, Exercise, Insulin resistance, Obesity, Public health, Physical activity

## Abstract

**Background:**

In terms of the potential influence of rapid weight loss (RWL) on the metabolic health of former combat sports athletes (CSA), the scientific literature is quite scarce. Therefore, the objective of the presented research was to determine the differences in metabolic syndrome (MetS) parameters and the prevalence of MetS between former athletes who performed RWL and athletes who did not.

**Methods:**

The sample of the presented study comprised 150 participants from Serbia, equally divided into two groups: 75 former athletes who had practiced combat sports and 75 ex-athletes of various other sports who did not practice RWL during their careers. The following parameters related to the MetS were evaluated: waist circumference, high-density lipoprotein cholesterol, systolic blood pressure, diastolic blood pressure, fasting glucose, and triglycerides. The oral glucose tolerance test (OGTT) was used to assess the participant's body response to sugar.

**Results:**

The RWL group had significantly higher both systolic (*p* < 0.001) and diastolic blood pressure (*p* < 0.001) compared to the group of athletes who did not practice weight reduction during their careers. Additionally, a tendency toward statistically significant differences between groups was recorded in the variable triglycerides (*p* = 0.069). Regarding OGTT, increased values of fasting blood glucose at the final measurement were revealed only in the RWL group (*p* = 0.003). The prevalence of MetS was substantially higher in CSA than in the control group (39.5% vs. 16.2%, respectively *p* = 0.002).

**Conclusions:**

This study suggests that former elite CSA who used RWL during their sports career are susceptible to negative metabolic alterations at the end of their competitive period.

**Supplementary Information:**

The online version contains supplementary material available at 10.1186/s12889-024-17763-z.

## Introduction

Lack of exercise and being overweight and obese are the causes of metabolic syndrome (MetS), which is currently assuming almost epidemic proportions [1]. Resistance and strength training have a positive influence on insulin resistance, stimulating the uptake of glucose by muscle cells [1]. Physical activity is considered very efficient regarding the effects on the metabolic profile of former athletes [2]. Physical exercise is also associated with many health-related benefits, reducing the risk of developing various chronic diseases, such as obesity, cardiovascular disease, and type II diabetes mellitus [3–5]. There is evidence to suggest that life-long physical activity may be an important factor for MetS prevention in adulthood, demonstrating the significant incidence of MetS among inactive males and females [6]. Finally, epidemiological studies suggest that former elite athletes have a lower prevalence of cardiovascular disease, diabetes, and hypertension compared to the general population [7, 8], emphasizing the importance of physical activity and a healthy lifestyle [9, 10].

Rapid weight loss (RWL) is considered a sports practice that can negatively affect the metabolic balance of athletes. Typically, RWL is defined as a 5% reduction in body weight less than one week before competition [11]. Rapid weight loss is often performed to compete in a lower weight category to gain a competitive advantage over rivals and have a greater opportunity to win the competition [12–16]. Furthermore, the methodologies used for RWL are varied, from gradual dieting, sauna use, and skipping meals, the most dominant ones to reduce weight before competition, up to more extreme methods of RWL, such as the use of laxatives, diuretics, diet pills and vomiting [17]. Therefore, alternative methods of sustainable weight loss should be available for athletes [17]. The interactive effect of food restriction and fluid deprivation may cause adverse physiological effects on the body [18]. This stress caused over several years could also lead to permanent adverse metabolic effects for these former athletes [19].

In the scientific literature, the term MetS is universally used [20]. Specifically, metabolic syndrome has been defined according to the International Diabetes Federation and the American Heart Association/National Heart, Lung, and Blood Institute [21] as a combination of at least 3 or more of the following factors: abdominal obesity (waist circumference (WC) > 102 cm in men); hyperglycaemia (fasting blood glucose (FBG) ≥ 100 mg/dL or current use of insulin or oral hypoglycaemic drugs); hypertriglyceridemia (triglycerides ≥ 150 mg/dL); low HDL cholesterol (HDL < 40 mg/dL in men); high blood pressure (BP) (systolic/diastolic BP ≥ 130/85 mmHg) or regular use of antihypertensive drugs. To define abdominal obesity, we have adopted the European Cardiovascular Society cut-off point for the European population [22, 23].

The United States and many other countries are facing an epidemic of insulin resistance that represents a risk factor for cardiovascular disease. This is occurring in the context of a marked increase in the number of individuals diagnosed with type 2 diabetes mellitus and a dramatic rise in obesity [24]. These MetS trends are likely to continue to increase over time across the global population [25].

Currently, there is a gap in the literature regarding the potential effects of RWL on the metabolic health of former combat sports athletes (CSA). Therefore, the objective of the presented research was to determine the differences in MetS parameters and MetS prevalence between former CSA who implemented RWL during their career and respondents from other sports who did not implement RWL. The authors hypothesized that MetS factors would be more pronounced in athletes who practiced RWL methods in their careers and that the greater prevalence of MetS would also be recorded in this group.

## Methods

### Experimental approach to the problem

This retrospective case–control study was performed in July 2023 at the Provincial Institute for Sports Medicine and Faculty of Sports and Physical Education, University of Novi Sad, Serbia. During the study, various measurements were conducted, including anthropometric parameters, FBG, lipid profile, and BP. The oral glucose tolerance test (OGTT) was administered by specialized medical personnel [26]. All examinations were performed in the morning, taking an FBG sample, followed by ingestion of 75g (standardized procedure) of glucose dissolved in 250–300 ml of water, in the shortest time possible. In the following hours, the patient was asked to remain seated, without smoking or eating, possibly relaxed (emotional stress could distort the results). Two hours later, a new measurement of basal glycemia was carried out and a comparison was subsequently made between the 2 measurements. Using FBG and 2-h glucose afterload, a possible impaired glucose tolerance is defined by a 2-h glucose afterload of 140 to 199 mg/dL or an FBG between 110 and 125 mg/dL.

### Participants and sample size

A priori sample size calculation was carried out using G*power 3.1.9.4. Based on the effect size of 0.50, alpha levels of 0.05, and a power of 0.80, a total of 148 respondents, divided into two groups of 74 former athletes, were required to detect between-group differences. Therefore, a total of 150 respondents were recruited for this investigation. Athletes were divided into two groups: RWL group (*n* = 75, mean age 51.53 ± 11.38 years, mean height 176.87 ± 6.42 cm, and mean weight 90.43 ± 16.06 kg), which encompassed CSA who competed in judo, jujitsu, karate, kickboxing, taekwondo, boxing and implemented RWL during their careers, and control group (CG) (*n* = 75, mean age 49.77 ± 15.02 years, mean height 181.64 ± 7.04, cm and mean weight 91.47 ± 14.59 kg) that represented athletes from various sports, such as swimming, soccer, mountain climbing, handball, athletics, rowing, and water polo and did not practice RWL during their carriers. In addition, to be included in the study, participants needed to meet the following eligibility criteria: to be elite male athletes and finish their sports career at least 10 years ago. Regarding RWL, data were collected using a questionnaire that assessed the history of weight reduction among former combat sports athletes. Only athletes who implemented RWL at least three times per year during their competitive careers, losing approximately 5% of their body weight less than one week before competition, were included in the investigation. This study was conducted in accordance with the Declaration of Helsinki [27] and ethical approval was obtained from the ethics committee of the University of Novi Sad, Serbia (Ref. No. 49–10-02–2023-1).

### Informed consent was obtained from all subjects and or legal guardians

#### Measurements

Anthropometric measurements included body height, body weight, body mass index (BMI), and WC. All measurements were conducted by trained researchers using standardized methods. Body height was measured without shoes with a wall-mounted stadium tape measure and a calibrated scale with an accuracy of 0.1 cm, while body weight was measured with a Seca professional scale (maximum recordable weight: 300 kg; resolution: 100 g; Seca, Hamburg, Germany). We calculated BMI (kg/m^2^) based on self-reported height (m) and weight (kg) and considered overweight and obesity if BMI was between 25 kg/m^2^ and 29.9 kg/m^2^ and ≥ 30 kg/m^2^, respectively. Additionally, WC was measured by wrapping a flexible tape measure around the bare abdomen, starting at the top of the hip bone at the level of the navel. It was ensured during the circumference measurement that the tape measure was not too tight and that it was straight. Blood samples were taken fasting state of participants in the morning. For glucose, a double measurement was carried out, including evaluation before starting the OGTT test and 2 h after the end of the test. Each blood sample was collected in special tubes without anticoagulant to obtain serum. The samples were centrifuged at 1300 × g for 15 min. Blood pressure was measured with a sphygmomanometer (Riester Diplomat, Jungingen, Germany) from the participant's right arm using a cuff measuring 14.5 × 54.5 cm after at least 5 min of rest in a sitting position. Blood pressure was measured twice and a pause of at least 1 min was taken between measurements. The average of two values was used in the calculations. The data regarding HDL cholesterol and triglycerides were collected in the laboratory following a blood sample from the arm of the participants who had followed a fasting period of at least 12 h. Then, the values were recorded in the appropriate forms. The International Physical Activity Questionnaire was administered to assess the physical activity performed by each respondent [28]. Post-career physical activity was calculated in line with the recommendations for adults from the American College of Sports Medicine and the American Heart Association [29]. Specifically, physical activity was considered lower than recommended (lower physical activity recommendation) if less than 30 min per day on 5 days per week of moderate-intensity aerobic activity or less than 20 min per day on 3 days per week of vigorous-intensity aerobic activity was performed at the end of the career. Conversely, if at least 30 min per day for 5 days per week of moderate-intensity aerobic activity or 20 min per day for 3 days per week of vigorous-intensity aerobic activity were performed the participant adhered to the physical activity recommendations (Meet physical activity recommendation). A second questionnaire (Sports Medicine Questionnaire) was administered to assess and categorize alcohol and cigarette use. Regarding alcohol consumption, we adopted the World Health Organisation guidelines [30]; classifying subjects as hazardous drinkers, moderate drinkers, or abstainers. In particular, those who have drunk 5 or more standard alcoholic drinks on a single occasion in the last 30 days are considered hazardous drinkers; if they did not drink 5 standard alcoholic drinks on a single occasion in the last 30 days they were considered moderate drinkers, and abstainers if they never had drunk alcohol. Current smoking status was divided into 3 categories, including current smoker, ex-smoker, or never smoker.

### Statistical analysis

All data were analyzed with SPSS (Statistical Package for Social Sciences ver. 24.0, IBM Statistics, Armonk, NY, USA). The normality of the distribution was checked using the Kolmogorov–Smirnov test. In terms of demographic and MetS parameters, the comparison between RWL and CG was performed using the Mann–Whitney test, while the Wilcoxon test examined differences for pre-post glycemia in both groups. The prevalence of overweight/obesity, the presence of MetS, and the differences in lifestyle variables between the analyzed groups were tested with the Chi-squared test. Further, the biserial rank correlation was utilized as a measure of the effects size, and interpreted as trivial (0–0.1), small (0.10–0.30), moderate (0.30–0.50), and large (≥ 0.50) [31]. Data were reported as mean, standard deviation, and 95% confidence interval for MetS variables. For the Chi-squared test, data were presented as percentages. The significance level was set at p ≤ 0.05.

## Results

Demographic and lifestyle parameters of participants are shown in Table 1. No differences between groups were observed regarding athletes' age and body weight. Contrary, statistically significant differences were recorded in the body height (176.87 ± 6.42 cm vs. 181.64 ± 7.05 cm, *p* = 0.001) and BMI (28.83 ± 4.43 vs. 27.60 ± 3.86, *p* = 0.04). The RWL group had substantially higher values of BMI relative to the athletes from the CG. There were no statistically significant differences between the analyzed groups in all lifestyle variables, including tobacco, alcohol, and physical activity.

**Table 1:** Demographic and lifestyle variables of respondents

Variables	RWL group	Control group	*P* values
Age (years)	51.53 ± 11.38	49.77 ± 15.02	0.144
Body height (cm)	176.87 ± 6.42	181.64 ± 7.05	0.001***
Body weight (kg)	90.43 ± 16.07	91.47 ± 14.60	0.58
BMI (kg/m^2^)	28.83 ± 4.43	27.60 ± 3.86	0.04*
**Tobacco (%)**			0.375
Never smokers	91.9	90.5	
Former smokers	5.4	2.7	
Current smokers	2.7	6.8	
**Alcohol (%)**			0.283
Abstainers	55.4	44.6	
Moderate drinkers	2.7	6.8	
Hazardous drinkers	41.9	48.6	
**Physical activity (%)**			0.569
Meet recommendations	27	23	
Lower than recommended	73	77	

*BMI* Body mass index, Statistical significance—***; *p* < 0.001—*; *p* < 0.05

Analyzing the parameters concerning the MetS, statistically significant differences were found for both systolic and diastolic BP (129.89 ± 12.03 mmHg vs 124.59 ± 12.39 mmHg, *p* = 0.001) and (81.58 ± 11.52 mmHg vs 75. 97 ± 7.67 mmHg, *p* = 0.001), respectively (Table 2). In addition, a tendency toward statistically significant differences was noted in a triglycerides variable (*p* = 0.069). However, there were no differences between the RWL group and the CG in parameters such as WC, HDL cholesterol, and FBG.

**Table 2:** Metabolic Syndrome parameters

Variables	RWL group	Control group	Mean differences (95% CI)	ES	*P* value
Waist circumference (cm)	104.78 ± 9.22	104.11 ± 6.94	0.66 (-1.97, 3.30)	0	0.973
Triglycerides (mg/dL)	31.86 ± 17.28	27.54 ± 15.66	4.32 (-1.00, 9.64)	0.17	0.069
HDL cholesterol (mg/dL)	23.22 ± 5.04	23.22 ± 4.68	0 (-1.57, 1.57)	0.02	0.826
GL120 (mg/dL)	112.86 ± 45.54	98.82 ± 29.7	14.04 (1.63, 26.45)	0.14	0.129
Systolic blood pressure (mmHg)	129. 89 ± 12.03	124.59 ± 12.39	5.3 (1.36, 9.24)	0.40	0.001***
Diastolic blood pressure (mmHg)	81.58 ± 11.52	75.97 ± 7.67	5.61 (2.45, 8.77)	0.29	0.001***

*ES* Effect size—*CI* Confidence interval, *GLI20* Glucose after OGTT test (2° measurement), *RWL* Rapid weight loss

Table 3 depicts the prevalence of overweight/obesity and MetS in both groups. A significant percentage of participants in each group were overweight or obese and no differences between them have been revealed. Nonetheless, the percentage of MetS was considerably higher in the RWL group than in the athletes who did not lose body weight during their career (39.5% vs. 16.2%, respectively *p* = 0.002).

**Table 3:** Prevalence of Metabolic Syndrome among athletes

	RWL group	Control group	*P* value
Overweight/obesity (%)	85.5	77	0.182
With metabolic syndrome (%)	39.5	16.2	0.002**

*RWL* Rapid weight loss—*P*-value; Statistical significance—**; *p* < 0.01

**Fig. 1:**
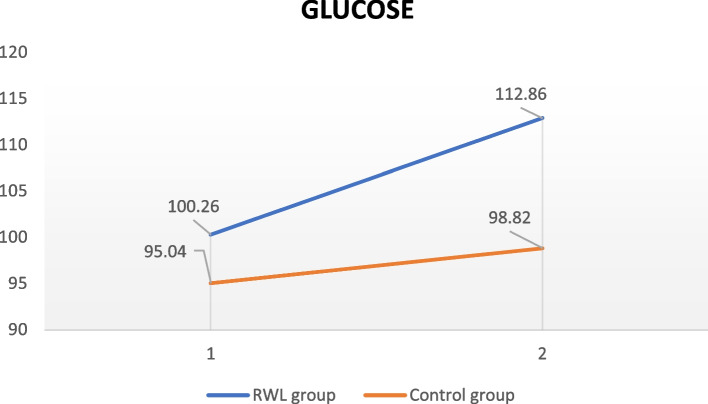
Glucose trend at first and second measurement Note: *RWL; Rapid weight loss– 1; first glucose measurement – 2; second glucose measurement (after 2 h)*

Figure 1 demonstrates the effects of the implemented intervention in both groups. Following OGTT, it is obvious that in the RWL group, the level of FBG was substantially higher compared to the baseline assessment (100.26 ± 21.6 mg/dL vs 112.86 ± 45.54 mg/dL, *p* = 0.003). On the other hand, no differences between final and initial measurements were observed in the CG.

## Discussion

The objective of the present research was to determine the differences in MetS parameters and the prevalence of MetS between former CSA and respondents who did not perform RWL during their sports careers. Our hypothesis is partially confirmed, since differences were detected between the two groups in 2 out of 5 variables regarding MetS, including systolic and diastolic BP. In addition, a significant trend toward differences was observed for triglycerides (*p* = 0.069). Most importantly, following the OGTT test, a substantial increase in FBG was found only in the RWL group. At last, a higher prevalence of MetS was recorded in the RWL group (39.5%) than in the group of respondents who did not lose body weight during their careers (16.2%).

The findings of the presented investigation related to the prevalence of MetS highlight a potential connection between RWL practices and the occurrence of this phenomenon among former CSA. These results are in accordance with previous studies that examined the relationship between RWL and several health variables of CSA [18, 32–35]. Namely, the negative health consequences are multiple, such as acute kidney damage [18, 34], significant muscle damage [32], and general injury due to training. Moreover, numerous studies defined additional detrimental influences of RWL, such as reduction in bone mineral mass, worsening of muscle performance, body dehydration, increased heart rate, and increased anxiety [19, 36, 37].

Another important aspect of this study is the analysis of OGTT. The results demonstrated substantial differences in FBG levels between the two groups. In particular, the group of athletes who practiced RWL during their careers experienced a significant increase in FBG levels in the second measurement after the OGTT. The results obtained suggest that former CSA who experienced RWL may be at increased risk of impaired glucose metabolism. The presented findings support the idea that RWL may have negative effects on insulin sensitivity and glucose regulation [38]. Additionally, it is noteworthy to emphasize that weight reduction negatively influences the level of hormones such as cortisol and testosterone [39, 40].

Numerous studies have analyzed the parameters of MetS and their prevalence in former athletes. Several parameters related to the MetS have been examined in literature in former athletes, and the results obtained highlighted lower HDL-cholesterol levels, increased blood pressure, and impaired FBG [41]. There is convincing evidence that former athletes who have been involved in endurance sports and vigorous training had a lower prevalence of MetS compared to former athletes who competed in sports such as soccer or American football [42]. At last, our results are inconsistent with a study in which 33% of 12 former professional footballers had MetS, compared to 50% in the CG [43]. This disagreement could be due to the different nature of the sport.

The practical implications of this study are of paramount importance for both current and former CSA. Athletes currently implementing RWL should be duly informed about the potential long-term risks associated with these practices. Furthermore, coaches, sports organizations, and medical professionals must prioritize the health of athletes by exploring alternative weight management methods to mitigate these risks caused by RWL [16]. Moreover, sports nutritionists could have a key role in monitoring the health of athletes and avoiding adverse events [44]. A truly worrying fact is that approximately 60% of judo athletes started performing RWL before competitions at a very early age (i.e. 12–15 years) [16]. The health consequences induced by RWL may be more pronounced in athletes who begin to lose weight at a very young age, putting them at greater risk of adverse health-related events in older age [16]. This could be one of the reasons that lead to adverse events in former CSA. The effect of nutritional and fluid limitation repeated during their career could lead to negative, sometimes permanent adverse metabolic effects for former CSA that practiced RWL [18]. In addition, dehydration induced by severe or moderate RWL increases the risk of acute cardiovascular problems [39], which may lead to MetS-related issues.

It is essential to emphasize the importance of maintaining a healthy lifestyle, including regular physical activity and proper nutrition, after retirement from sport. This attitude during the transition from athlete to former athlete can significantly contribute to reducing the risk of MetS and related diseases [45, 46]. Furthermore, additional research is needed to understand in more detail the mechanisms underlying the metabolic alterations associated with RWL. This could involve analyzing the specific effects of different RWL methodologies and their accumulation over time. Longitudinal studies, which monitor the metabolic health of CSA over an extended period after retirement, represent a key future direction. These studies could provide valuable information on the persistence and evolution of MetS and related conditions over time.

Several limitations are necessary to acknowledge, and they must be considered during the interpretation of the highlighted evidence. Namely, the presented investigation addressed only male athletes. Thus, more studies with the female population are warranted in the future. Moreover, the absence of a group of non-athletes is apparent. Lastly, due to the nature of the study, it was not possible to randomize the participants. In the future, it would be useful to conduct a randomized controlled trial related to the RWL and metabolic health of former CSA.

## Conclusion

In conclusion, this study showed that former elite CSA, who practiced RWL as part of their competitive period, may be at increased risk of metabolic alterations after their sporting career. Combat sports athletes should also carefully use RWL during their career, or it is recommended to rule it out completely due to the possible negative influence on the athletes' metabolic health. In addition, the improvement of a healthy lifestyle, including regular exercise and an adequate diet, are essential to prevent these negative consequences at the end of a competitive career. Further research is needed to fully and deeply understand the impact of RWL on the metabolic health of former CSA and to develop strategies to mitigate its long-term metabolic consequences.

### Supplementary Information

**Additional file 1. **GPower Analysis.

## Data Availability

The complete data used throughout the study are available from the corresponding author upon reasonable request.
