# Supplementary figures and images for: Prevalence of metabolic syndrome and its association with rapid weight loss among former elite combat sports athletes in Serbia
Source: BMC Public Health. 2024 Jan 22;24:245. doi: 10.1186/s12889-024-17763-z (PMC10801998; doi:10.1186/s12889-024-17763-z)

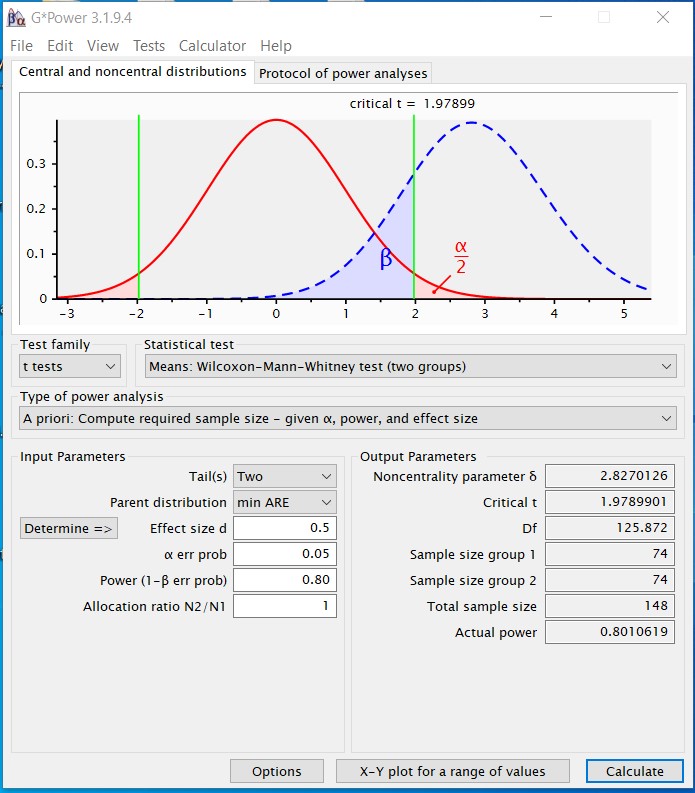

Supplement: Supplementary file 1 — Additional file 1. GPower Analysis. [file 12889_2024_17763_MOESM1_ESM.jpg]
